# Supplementary material for: Dynamic transcriptomic profiles of zebrafish gills in response to zinc supplementation
Source: BMC Genomics. 2010 Oct 11;11:553. doi: 10.1186/1471-2164-11-553 (PMC3091702; doi:10.1186/1471-2164-11-553)
Supplement: Additional file 2 — Interactive Direct Interaction Network representing the molecular interactions between zinc, copper, iron, calcium and proteins encoded by transcripts changed by zinc supplementation. Mini web-site containing index.html and hyperlinked pages in subdirectory describing a Direct Interaction Network automatically generated based on curated interactions contained within the proprietary PathwayArchitect database. Ovals represent proteins and the circles symbolize metal ions. Objects are coloured by their abundance in zebrafish at the time-point they were significantly different from the control is a scale from -4 fold (dark green) to +4 fold (dark red). Where significant differences were found at more than one time-point, the colour overlay shows expression at the first instance. Dark blue squares denote 'binding', and light blue squares 'expression'; green squares stand for 'regulation', green diamonds for 'metabolism', and green circles for 'promoter binding'. Arrow heads indicate directionality of the interaction where annotated. All nodes and edges can be further interrogated by selecting the relative area of the image. [file 1471-2164-11-553-S2.zip › PathwayArchitect Zn xs DIN/482954.html]

# BINDING:

|  |  |
| --- | --- |
| Type | BINDING |
| Effect | None |


---

|  |  |
| --- | --- |
| Score | 0 |


---

|  |  |
| --- | --- |
| Reference Count | 6 |


---

|  |  |
| --- | --- |
| Mechanism | Unknown |


---

|  |  |
| --- | --- |
| Reference:0 || Sentence | "Present results show that CO and O2 bindings to the heme iron of Mb are controlled by Leu29(B10) by influencing the structure of close vicinity of the heme and the geometry of iron-bound ligand." |
| PMID | 1618766 |
| Year | 1992 |
| Species | Human |
| Journal | J Biol Chem |
| RefScore | 1 |
| Source | PArchNLP |
  |
|  |  |


---

|  |  |
| --- | --- |
 Reference:1 || Sentence | "The M�ssbauer effect of 57Fe-enriched samples was used to investigate the coupling of 80% sucrose/water, a protein-stabilizing solvent, to vibrational and diffusive modes of the heme iron of CO-myoglobin." |
| PMID | 9876153 |
| Year | 1999 |
| Journal | Biophys J |
| RefScore | 0 |
| Source | PArchNLP |
  ||  |  |


---

|  |  |
| --- | --- |
 Reference:2 || Sentence | "The causes of the strong coupling of the iron-histidine vibration to the Soret resonance in the resonance Raman spectra of deoxyhemoglobin, myoglobin, and peroxidase are explored, using the vibronic theory." |
| PMID | 8298023 |
| Year | 1993 |
| Journal | Biophys J |
| RefScore | 2 |
| Source | PArchNLP |
  ||  |  |


---

|  |  |
| --- | --- |
 Reference:3 || Sentence | "Myoglobin, an intracellular iron containing protein that binds oxygen reversibly, has been shown in model systems to facilitate the diffusion of oxygen and thereby maintain the mechanical function of exercising canine skeletal muscle and of hypoxic benthic fish hearts." |
| PMID | 3791352 |
| Year | 1986 |
| Journal | Cardiovasc Res |
| RefScore | 0 |
| Source | PArchNLP |
  ||  |  |


---

|  |  |
| --- | --- |
 Reference:4 || Sentence | "The interaction of ascorbate with different heme iron redox states of myoglobin (ferrylmyoglobin, FeIV = O; metmyoglobin, FeIII; and oxymyoglobin, FeIIO2) was examined by e.s.r. and absorption spectroscopy." |
| PMID | 8405472 |
| Year | 1993 |
| Journal | FEBS Lett |
| RefScore | 1 |
| Source | PArchNLP |
  ||  |  |


---

|  |  |
| --- | --- |
 Reference:5 || Sentence | The reaction of small ligands within the distal pocket of haem proteins such as myoglobin, to form ligated, low-spin iron complexes is an archetypal spin-forbidden process in bioinorganic chemistry, because the initial, "deoxy" iron complex has a high-spin ground state. |
| PMID | 15471345 |
| Year | 2004 |
| Journal | Faraday Discuss |
| RefScore | 0 |
| Source | PArchNLP |
  |


---

|  |  |
| --- | --- |
